# Supplementary material for: A whole-animal phenotypic drug screen identifies suppressors of atherogenic lipoproteins
Source: eLife. 2026 Aug 27;14:RP105314. doi: 10.7554/eLife.105314 (PMC13521566; doi:10.7554/eLife.105314)
Supplement: Supplementary file 2 [file elife-105314-code1.zip › JHDL_primary_result/GENTIAN VIOLET/GENTIAN VIOLET_JHDL_3_017_018_2019_04_26_12_48.pdf]

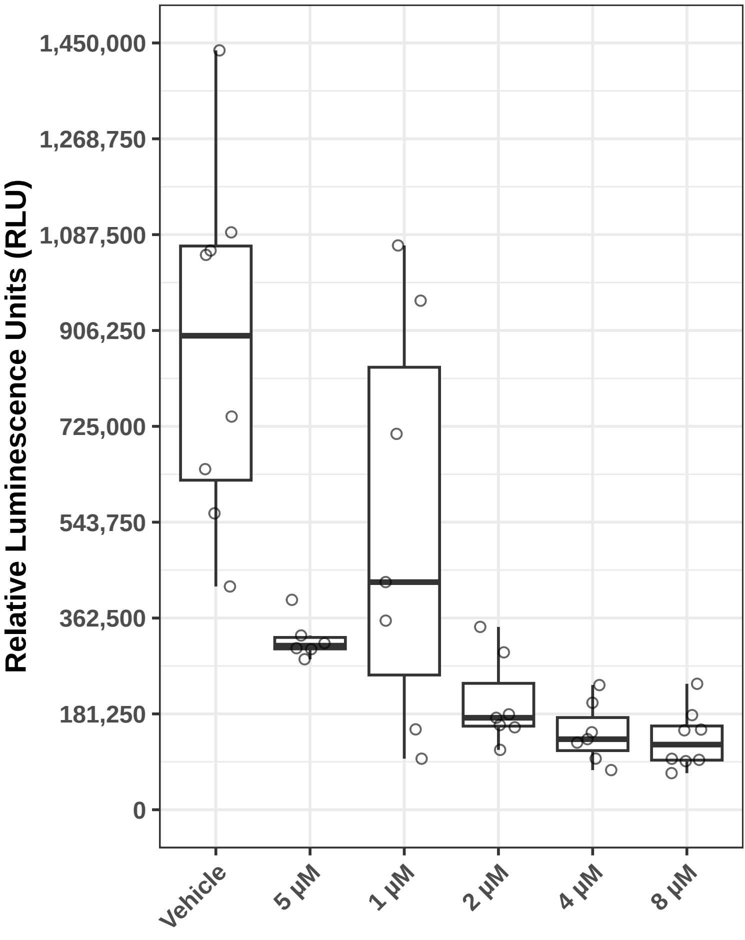

Relative Luminescence Units (RLU)
1,450,000
1,268,750
1,087,500
906,250
725,000
543,750
362,500
181,250
0
Vehicle
5 µM
1 µM
2 µM
4 µM
8 µM
